# Role of Neural NO Synthase (nNOS) Uncoupling in the Dysfunctional Nitrergic Vasorelaxation of Penile Arteries from Insulin-Resistant Obese Zucker Rats

**DOI:** 10.1371/journal.pone.0036027

**Published:** 2012-04-23

**Authors:** Ana Sánchez, Cristina Contreras, María Pilar Martínez, Belén Climent, Sara Benedito, Albino García-Sacristán, Medardo Hernández, Dolores Prieto

**Affiliations:** 1 Departamento de Fisiología, Facultad de Farmacia, Universidad Complutense de Madrid, Madrid, Spain; 2 Departamento de Anatomía y Anatomía Patológica Comparadas, Facultad de Farmacia, Universidad Complutense de Madrid, Madrid, Spain; Istituto Dermopatico dell'Immacolata, Italy

## Abstract

**Objective:**

Erectile dysfunction (ED) is considered as an early sign of vascular disease due to its high prevalence in patients with cardiovascular risk factors. Endothelial and neural dysfunction involving nitric oxide (NO) are usually implicated in the pathophysiology of the diabetic ED, but the underlying mechanisms are unclear. The present study assessed the role of oxidative stress in the dysfunctional neural vasodilator responses of penile arteries in the obese Zucker rat (OZR), an experimental model of metabolic syndrome/prediabetes.

**Methods and Results:**

Electrical field stimulation (EFS) under non-adrenergic non-cholinergic (NANC) conditions evoked relaxations that were significantly reduced in penile arteries of OZR compared with those of lean Zucker rats (LZR). Blockade of NO synthase (NOS) inhibited neural relaxations in both LZR and OZR, while saturating concentrations of the NOS substrate L-arginine reversed the inhibition and restored relaxations in OZR to levels in arteries from LZR. nNOS expression was unchanged in arteries from OZR compared to LZR and nNOS selective inhibition decreased the EFS relaxations in LZR but not in OZR, while endothelium removal did not alter these responses in either strain. Superoxide anion production and nitro-tyrosine immunostaining were elevated in the erectile tissue from OZR. Treatment with the NADPH oxidase inhibitor apocynin or acute incubation with the NOS cofactor tetrahydrobiopterin (BH4) restored neural relaxations in OZR to levels in control arteries, while inhibition of the enzyme of BH4 synthesis GTP-cyclohydrolase (GCH) reduced neural relaxations in arteries from LZR but not OZR. The NO donor SNAP induced decreases in intracellular calcium that were impaired in arteries from OZR compared to controls.

**Conclusions:**

The present study demonstrates nitrergic dysfunction and impaired neural NO signalling due to oxidative stress and nNOS uncoupling in penile arteries under conditions of insulin resistance. This dysfunction likely contributes to the metabolic syndrome-associated ED, along with the endothelial dysfunction also involving altered NO signalling.

## Introduction

Erectile dysfunction (ED) is currently considered as an early clinical sign of vascular disease due to its high prevalence in patients with cardiovascular risk factors including diabetes, hypertension and hyperlipidemia [1], [2]. ED is a common complication and an important cause of decreased quality of life in men with diabetes, male impotence being three times more prevalent in type 1 and type 2 diabetic patients than in the general population [3], [4].

Penile erection is a complex and multifactorial hemodynamic process involving organic pathways that require hormonal balance and neuronal and vascular function integrity [5], [6]. Erection is initiated by activation of parasympathetic nerves upon sexual stimulation, neural signs from the spinal cord stimulating neuronal nitric oxide synthase (nNOS) activity and NO production from non-adrenergic non-cholinergic (NANC) nerve terminals thereby causing an increase in blood flow to the cavernosal tissue [5], [6], [7]. Endothelial NOS (eNOS) is then activated by the continued shear stress on the endothelial lining of the sinusoidal spaces and arteries, which continues to produce endothelial-derived NO thus maintaining the sustained phase of penile erection [6], [7], [8].

Diabetic patients have an increased risk of vascular and nerve dysfunction and both autonomic neuropathy and endothelial dysfunction are considered the main etiological factors in the diabetic ED [9], [10]. Hyperglycemia, oxidative stress and altered lipid profiles contribute to vascular complications including peripheral nerve perfusion deficits which play an important role in the etiology of diabetic neuropathy [11]. Impairment of the NO-mediated neural and endothelial relaxations was first demonstrated as a cause of ED in human erectile tissue from diabetic patients [12]. Since then, several i*n vitro* and *in vivo* studies in animals and humans have demonstrated that a dysfunctional nitrergic system underlies the pathophysiology of ED in diabetes thus explaining the origin of diabetic impotence [12], [13], [14], [15]. Thus, neuronal and endothelial NO-dependent cavernosal smooth muscle relaxations are diminished in animal models of type 1 diabetes-associated ED [13], [15], [16]. Likewise, decreased erectile responses to pelvic nerve stimulation and impaired NANC nerve-mediated relaxations in cavernosal tissue have been found in prediabetic obese Zucker rats (OZR) [17] and in type 2 diabetic db/db mice, respectively [18], [19]. In contrast, enhanced corporal NANC-mediated relaxations despite impaired erectile function have been reported in Goto-Kakizaki non-obese type 2 diabetic rats and suggested as a compensatory mechanism to overcome restricted pre-penile arterial blood supply [20].

Although impairment of the NO-mediated vasodilator responses is well established, the mechanisms of the nitrergic defect underlying the diabetes-associated ED are not well understood and reduced NOS expression levels and activity [14], selective degeneration of nitrergic nerves to the penis [15], [21] and altered NO signalling [20], [22] have been proposed. We have recently demonstrated endothelial dysfunction and changes in the NO signalling in the penile endothelium of the OZR [23], [24], an established model of metabolic syndrome/prediabetes-associated ED [17].

The aim of the present study was to assess whether impairment of the neural NANC nitrergic relaxations may also contribute to the diminished erectile function in this model, and if so, to clarify the mechanisms involved in the nitrergic dysfunction with special reference to the role of oxidative stress and changes in the NO signalling.

## Materials and Methods

### Ethics Statement

The investigation conformed to the European Union Guidelines for the Care and the Use of Laboratory Animals (European Economic Community Directive 86/609/EEC) and all the experimental protocols were approved by the Institutional Animal Care and Use Committee of Madrid Complutense University.

### Animal model

Male OZR (*fa/fa,* n = 25) and their control counterparts, Lean Zucker rats (LZR) (*fa/−,* n = 25) were purchased from *Charles River Laboratories* (Barcelona, Spain) at 8–10 weeks of age. Animals were housed at the Pharmacy School animal care facility and maintained on standard chow and water ad libitum until they were used for study, at 17–19-weeks age.

### Dissection and mounting

Rats were killed by cervical dislocation and exanguination. The penile arteries, first- or second order branches of the rat dorsal penile artery from LZR and OZR rats were carefully dissected by removing the connective and fat tissue, as described previously [23]. Segments of dorsal penile arteries were cut into ring segments and mounted in parallel in double microvascular myographs (Danish Myotechnology, Denmark) by inserting two 40 µm tungsten wires into the vessel lumen. After mounting the arteries were equilibrated for 30 min in Krebs solution mantained at 37°C of the following composition (mM): NaCl 119, NaHCO_3_ 25, KCl 4.7, KH_2_PO_4_ 1.17, MgSO_4_ 1.18, CaCl_2_ 1.5, ethylenediaminetetraacetic acid 0.027 and glucose 11, continuously gassed with a mixture of 5% CO_2_/95% O_2_ to maintain pH at 7.4. The relationship between passive wall tension and internal circumference was determined for each individual artery and from this, the internal diameter, l_1_, that yielded a circumference equivalent to 90% of that given by an internal pressure of 100 mmHg was calculated [23].

### Experimental procedure

Electrical field stimulation (EFS) was performed through two electrodes mounted parallel to the vessel segments using a Cibertec stimulator (Barcelona, Spain) with constant current output. Squared pulses of 0.3 ms duration in 20 s trains with varying frequencies (0.5 to 16 Hz) were applied. Voltage of the stimulator was adjusted to deliver 35 mA. Changes on isometric force were recorded. At the beginning of each experiment, arteries were challenged once with 120 mM K^+^ (high K^+^-physiological saline solution, KPSS) in order to verify the contractile ability of the preparations. To assess the relaxant responses to nerve stimulation under NANC conditions, penile arteries from LZR and OZR were incubated with guanethidine (10^−5^ M) plus atropine (10^−6^ M), to inhibit adrenergic and cholinergic responses, respectively, and these drugs were kept present throughout the experiment to block NANC-mediated responses. To evaluate α-adrenergic nerve-mediated responses, EFS curves (0.5 to 32 Hz) were obtained on resting tension in the presence of the β-adrenergic blocker propranolol (10^−6^ M) and in the absence and presence of the non-selective NOS inhibitor N^G^-nitro-L-arginine (L-NOARG, 10^−4^ M). The role of the vascular endothelium was examined in arteries where the endothelium was mechanically removed by inserting a human hair in the vessel lumen and guiding it back and forwards several times. The absence of functional endothelium was confirmed by lack of the relaxation to acetylcholine (10^−5^ M).

A first frequency-response EFS curve (0.5 to 16 Hz) was obtained in phenylephrine (Phe) (10^−6^ M) precontracted arteries to compare LZR and OZR penile arteries relaxations. The effects of the NOS inhibitor L-NOARG (10^−4^ M), of the substrate of NO synthesis, L-arginine (3×10^−3^ M) and of L-arginine plus L-NOARG were tested before a second and third EFS curves were performed. The effects of mechanical removal of the endothelium, the selective inhibition of nNOS with N^W^-propyl-L-arginine hydrochloride (L-NPA, 3×10^−6^ M), scavengers of superoxide such as the superoxide dismutase (SOD) mimetic tempol (3×10^−5^ M) and the inhibitor of the nicotinamide adenine dinucleotide phosphate oxydase (NADPH) apocynin (10^−4^ M) were also assessed on the relaxations induced by EFS under NANC conditions. Treatment of penile arteries with the NOS cofactor tetrahydrobiopterin (BH4, 10^−4^ M), with its inactive stereoisomer (6R,S)-5,6,7,8-tetrahydro-D-neopterin dihydrochloride (NH4, 10^−5^ M) as a negative control, and with the inhibitor of the enzyme for BH4 synthesis GTP-cyclohydrolase, 2,4-diamino-6-hydroxypirimidine (GCH-I, 3×10^−4^ M) were also tested on the EFS-induced relaxations. When antagonists or inhibitors were used, drugs were introduced 30 minutes before a second concentration- or frequency-response curve was performed, and the Phe concentration was adjusted to match the contraction during the first control curve.

To assess the endothelium-independent relaxant responses, cumulative concentration-response curves (CCR) to the NO donor S-nitroso-N-acetylpenicillamine, SNAP (10^−8^–10^−4^ M) were obtained in Phe (10^−6^ M) pre-contracted penile arteries from LZR and OZR in the presence or the absence of SOD mimetic tempol (3×10^−5^ M) or the NADPH oxydase inhibitor, apocynin (10^−4^ M).

### Immunohistochemistry

Tissue samples from the penis containing the dorsal penile artery were inmersion-fixed in 4% paraformaldehyde in 0.1 M sodium phosphate-buffer (PB), cryoprotected in 30% sucrose in PB and snap frozen in liquid nitrogen and stored at −80°C. Transversal sections of 10 µm were obtained by means of a cryostat and processed following the avidin-biotin-peroxidase complex (ABC) method [25]. Sections were first immersed in a mixture of 1% H_2_O_2_ and 90% methanol in distilled water for 30 min, washed in PB and the pre-incubated in 10% normal goat serum in PB containing 0.3% Triton-X-100 for 2–3 h. Then, sections were incubated with a rabbit polyclonal antibody to the N-terminus of neuronal nitric oxide synthase (nNOS) (AB5380 Chemicon International Inc) diluted 1∶500 or with rabbit polyclonal antibody to Nitro-tyrosine (ab42789 abcam) diluted 1∶100 for 48 h at 4°C. Sections were then incubated for 2 h at room temperature with biotinylated anti-rabbit serum raised in goat (Chemicon International Inc) diluted 1∶400, followed by incubation with avidin-biotin-complex (ABC, Vector) 1∶100 dilution for 90 min at room temperature. The immunocomplex was visualized with 0.05% 3.3 diaminobenzidine (DAB) and 0.001% in H_2_O_2_ in PB. No immunoreactivity could be detected in sections incubated in the absence of the primary antisera.

### Measurement of superoxide production by chemiluminescence

Changes in the basal levels of superoxide were detected in the corpus cavernosum by lucigenin-enhanced chemiluminescence, as previously described in erectile tissue [26]. Corpora cavernosa (4–5 mm long strips) from LZR and OZR were dissected and equilibrated in Krebs buffer for 30 minutes at room temperature and then incubated in the presence or absence of the ROS scavenger, tempol (3×10^−5^ M) for 30 min at 37°C. The corpus cavernosum was then transferred to microtiter plate wells containing 5×10^−6^ M lucigenin (bis-N-methylacridinium nitrate) in air-equilibrated Krebs solution buffered with 10^−2^ M HEPES-NaOH, in the absence (controls) and presence of tempol. Chemiluminescence was measured in a luminometer (BMG Fluostar Optima), and for calculation baseline values were subtracted from the counting values under the different experimental conditions and superoxide production was normalized to tissue weight.

### Measurements of intracellular Ca^2+^ ([Ca^2+^]_i_)

Simultaneous measurements of [Ca^2+^]_i_ and force were performed in intact penile arteries as previously described [27]. The myograph was mounted on an inverted microscope (Axiovert S100 TV) equipped for dual excitation wavelength microfluorimetry. Penile arteries were incubated in the dark in PSS with 8 µM Fura 2-acetoxymethyl ester for 3 hours at 37°C, changing the solution after 1.5 h. Then arteries were washed for 45 minutes, to remove remaining Fura-2 acetoxymethy ester. After loading, the arteries were illuminated with alternating 340 and 380 nm light using a monochromator-based system (Deltascan, PTI). The fluorescence emission was detected at a wavelength of 510 nm. At the end of each experiment, Ca^2+^-insensitive signals were determined after quenching with 25 mM Mn^2+^, and the values obtained were substracted from those obtained during the experiment. The Ratio (R) F_340_/F_380_ was taken as a measure of [Ca^2+^]_i_.

Arteries were initially stimulated with KPSS in order to test vessel viability. Then, cumulative concentration-response curves to the NO donor SNAP (10^−8^–10^−4^ M) were evaluated in arteries from LZR and OZR pre-contracted with Phe (10^−6^ M).

### Data presentation and statistical analysis

Results are expressed as either Nm^−1^ of tension or as a percent of the response to either Phe or KPSS in each artery, as means ± SEM of 6–10 arteries (1–2 from each animal). Sensitivity of the arteries to the relaxant agonists or EFS is expressed in terms of EF_50_ or EC_50_ values, where pEF_50_ is −logEF_50_ and pEC_50_ is −logEC_50_, EF_50_ or EC_50_ are the frequency or the concentration of the agonist, respectively, required to produce 50% of the response and was calculated by non-linear curve fitting of the frequency-response curves for the inhibitor to the classical Hill Equation by using a nonlinear interactive fitting program (Graph Pad Prism 5.0, Graph Pad Software Inc., San Diego, CA, USA). The EF_50_ or EC_50_ value for each individual curve was first obtained and thereafter the average value for a given set of experiments was calculated. Statistically significant differences between means were analysed by one-way ANOVA or using paired or unpaired Student's *t* test when apropiate. Probability levels smaller than 5% were considered significant.

## Results

### General Parameters

At the time of the experiment (17–18 weeks of age), OZR were significantly heavier than LZR (518±7 g and 383±5 g, respectively, *P*<0.0001, n = 25). We have reported that at this age animals from the OZR group exhibit mild hyperglycaemia, hyperinsulinemia and dyslipidemia with elevated total cholesterol and triglycerides levels [23]. The normalized internal lumen diameters, l_1,_ were significantly smaller in penile arteries from OZR compared with LZR (124±4 µm, n = 49, and 144±4 µm, respectively, n = 52, *P*<0.001). The contractions to KPSS were reduced in the OZR group (1.2±0.1 N/m, n = 49) compared with LZR (1.8±0.13 N/m, *P*<0.001; n = 52), indicating altered contractility of arterial smooth muscle.

### NO-mediated neurogenic relaxations in penile arteries from LZR and OZR

**Figure 1:**
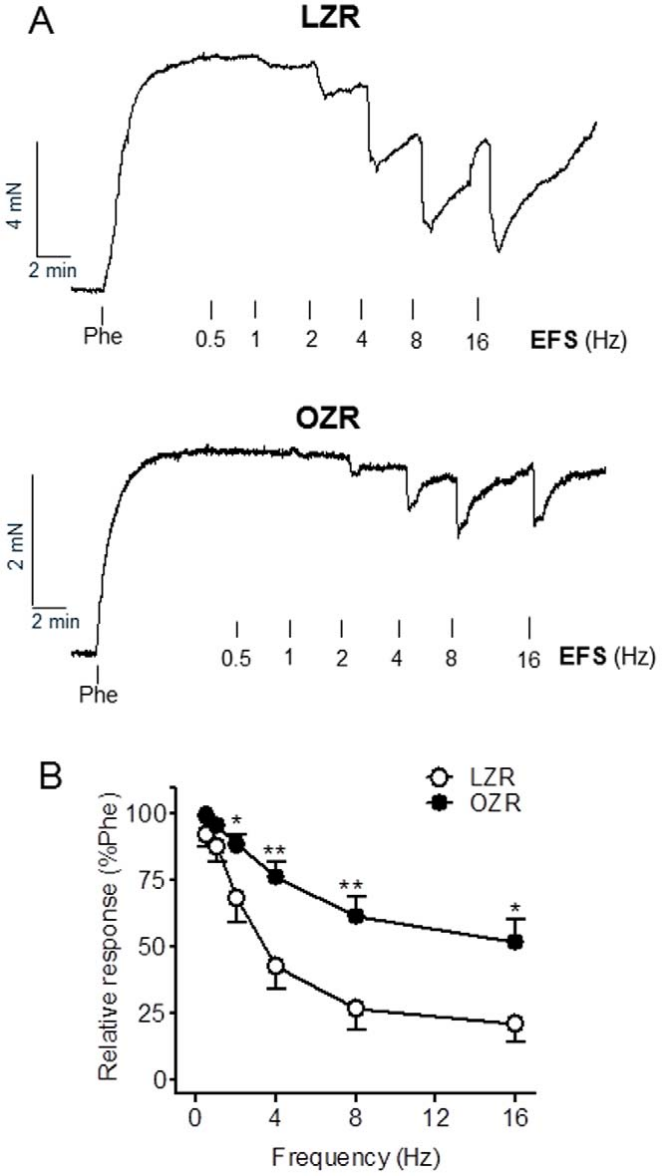
NANC vasorelaxant responses were impaired in penile arteries from OZR. **A.** Representative traces showing the relaxant responses induced by EFS (0.5–16 Hz) in penile arteries precontracted with Phe (10^−6^ M) under non-adrenergic non-cholinergic (NANC) conditions, which were significantly impaired in OZR compared with LZR. **B.** Average frequency-response curves for the relaxation to EFS in penile arteries from LZR and OZR. Data are shown as the means ± SEM of 7 and 9 arteries (1–2 per animal). * P<0.05, ** P<0.01 *vs* LZR.

An impaired neurogenic vasodilator response has been reported in several models of diabetes-associated ED. Nitrergic inhibitory neurotransmission was assessed in penile arteries from LZR compared to OZR. Under NANC conditions, EFS (0.5 to 16 Hz) induced frequency-dependent relaxations in arteries pre-contracted with Phe which were significantly reduced in OZR compared with LZR (Figures 1A and B). Thus EF_50_ were 4.89±0.48 (n = 8) and 3.14±0.40 (P<0.005, n = 7) in LZR and OZR, respectively, and high frequency stimulation (4 to 16 Hz) produced remarkably greater relaxant effects in healthy arteries compared with arteries from OZR (Figure 1B). This suggests an impaired inhibitory neurotransmission in penile arteries from obese animals.

**Figure 2:**
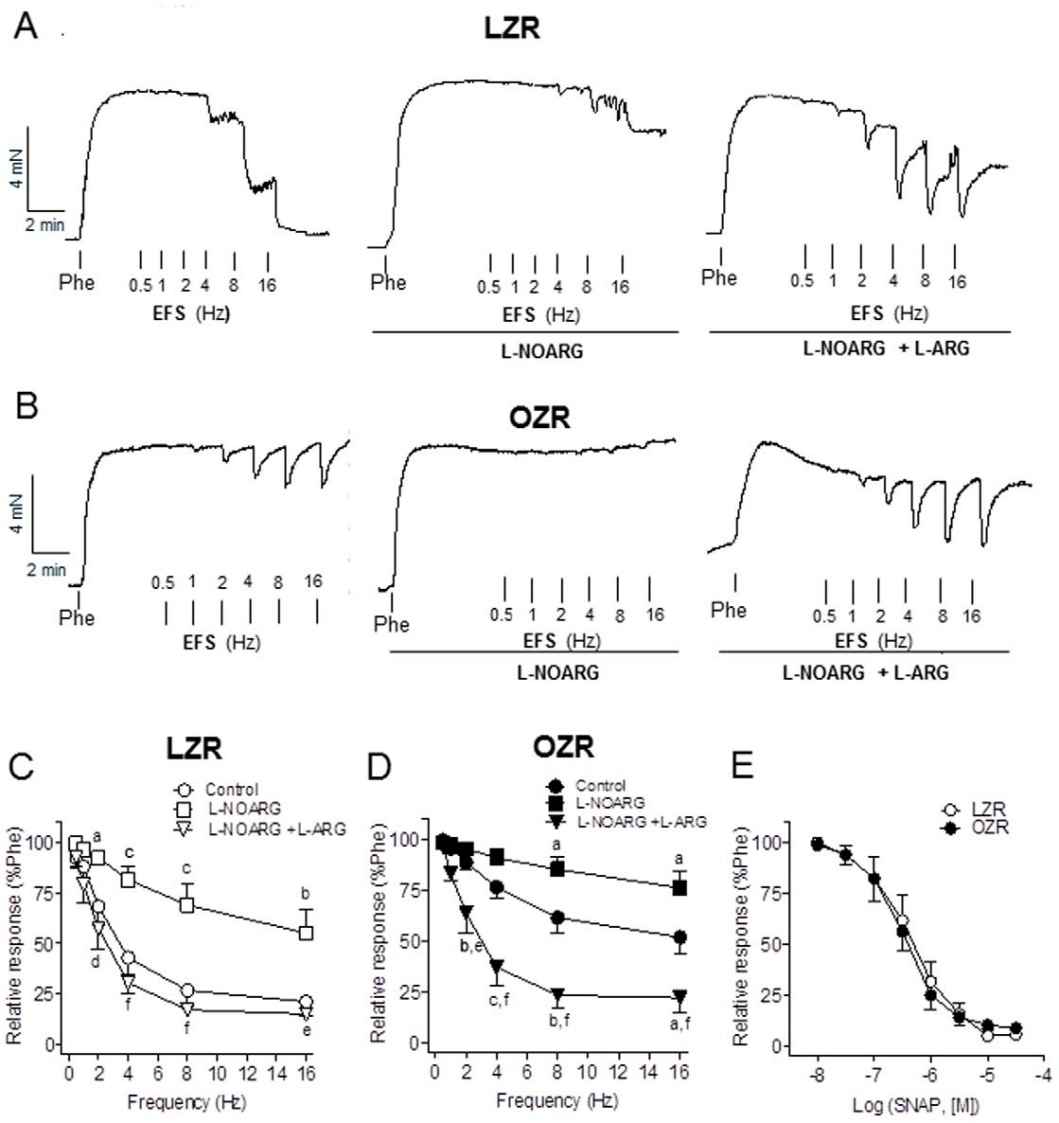
NO-mediated neural vasorelaxant responses were impaired but the relaxant responses to the NO donor SNAP were unaltered in penile arteries from OZR. **A, B.** Representative traces showing the effects of the NOS inhibitor (L-NOARG, 10^−4^ M) and of L-NOARG plus the substrate of the NO synthesis L-arginine (L-arginine, 3×10^−3^ M) on the EFS frequency- dependent relaxations (0.5–16 Hz) in penile arteries from LZR and OZR. Acute treatment with L-arginine restored neural NO-mediated response in OZR to control levels in LZR. **C, D.** Average frequency-response curves for the effects of NOS blockade and L-arginine on the relaxations to EFS in penile arteries from LZR and OZR. **E.** Relaxant responses to the NO donor SNAP were unaltered in penile arteries from OZR. Concentration-response curves for the relaxant effect of SNAP (10^−8^–10^−4^ M) in penile arteries from LZR and OZR. Results are means ± SEM of 6–8 arteries. ^a^P<0.05, ^b^P<0.01, ^c^P<0.001 *vs* control; ^d^P<0.05, ^e^P<0.01, ^f^P<0.001 *vs* L-NOARG-treated.

In order to verify whether the EFS relaxant responses are specific of nitrergic nerves, the effects of inhibitors and substrates of NO synthesis were tested. Treatment with L-NOARG (10^−4^ M) markedly inhibited the EFS-induced relaxations in penile arteries from LZR and OZR (Figure 2). Given acutely, the substrate of NO synthesis L-arginine (3×10^−3^ M) significantly reversed the inhibition induced by L-NOARG in both groups (Figures 2A and B), this effect of L-arginine being larger in arteries from obese animals where NO-dependent relaxations were restored to levels similar to those in control arteries (Figure 2D). These results indicate that the vasodilatation induced by EFS in penile arteries during erection is largely mediated by NO production and impaired in OZR. In contrast to the NO-mediated neurogenic responses, no significant differences were observed in the relaxations elicited by the NO donor SNAP between LZR and OZR (Figure 2E), pEC_50_ values being 6.30±0.21 (n = 7) in LZR, and 6.39±0.18 (n = 7; *P*>0.05) in OZR. These results suggest that the reduced NO neurogenic responses are due to impaired NO bioavailability in penile arteries from obese rats.

**Figure 3:**
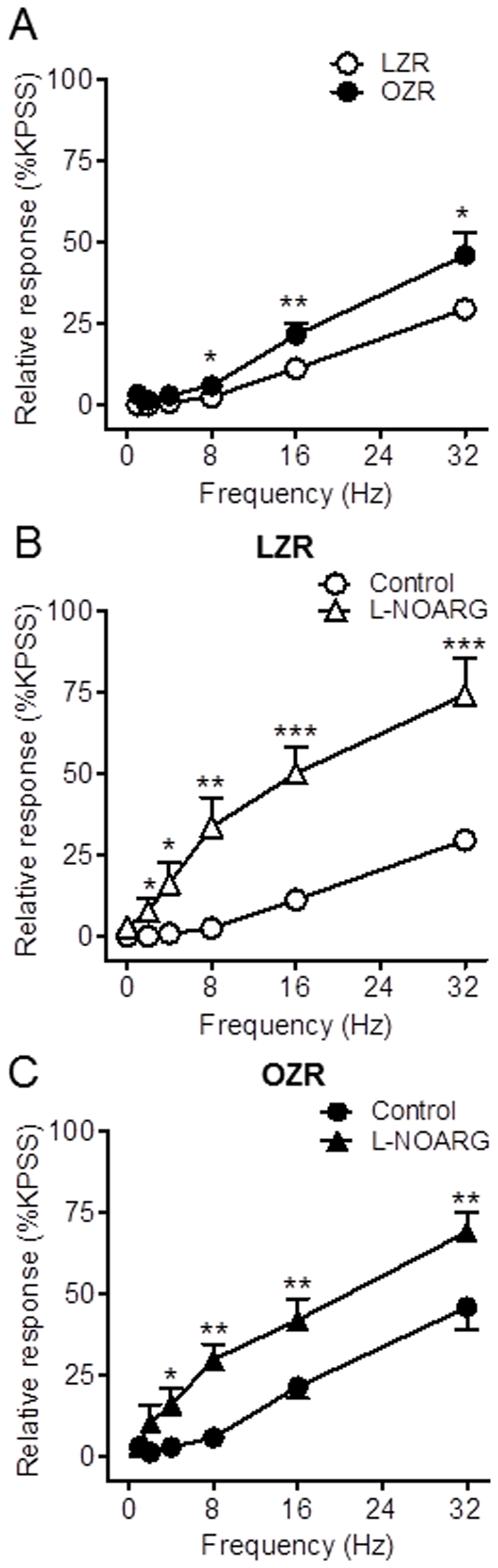
Neural vasoconstrictor responses were augmented in penile arteries from OZR in part due to impaired NO release. **A.** Average EFS frequency- dependent contractions (1–32 Hz) in penile arteries from LZR and OZR. **B, C.** Inhibition of NO synthesis largely enhanced neural vasoconstrictor responses to EFS agonists in LZR and to a lesser extent in OZR. Effects of the NOS inhibitor L-NOARG (10^−4^ M) on EFS frequency- dependent contractions in penile arteries of LZR and OZR. Results are mean ± SEM of 6–8 arteries. *P<0.05, **P<0.01, ***P<0.001 *vs* control.

Considering that the penis is kept in the flaccid state mainly by sympathetic activation and the release of vasoconstrictor noradrenaline, we next assessed the role of NO on the EFS-induced adrenergic contractions. In the presence of propranolol (10^−6^ M) to inhibit β-adrenergic receptors, EFS elicited frequency-dependent contractions that were abolished by the adrenergic toxin guanethidine (not shown) and significantly enhanced in penile arteries from OZR (Figure 3A), suggesting an augmented sensibility to α-adrenergic stimuli. Thus, EF_50_ values were18.5±0.8 (n = 7) and 17.5±1.3 (n = 7; *P*>0.05), and maximum effect 29±3% (n = 7) and 46±7% (n = 7; *P*<0.05) of the KPSS induced contraction, in LZR and OZR, respectively. EFS-induced vasoconstriction was potentiated by treatment with the NOS blocker L-NOARG (Figures 3B and C), this potentiation being lesser in penile arteries from OZR (Figure 3C). Thus, the contractile response induced by EFS at 16 Hz was enhanced after NOS blockade by 4.5- and 2-fold in LZR and OZR, respectively. These results indicate that under basal conditions NO modulates penile adrenergic vasoconstriction and confirm a reduced NO bioavailability in arteries from obese animals.

### nNOS-containing nerves in penile arteries

**Figure 4:**
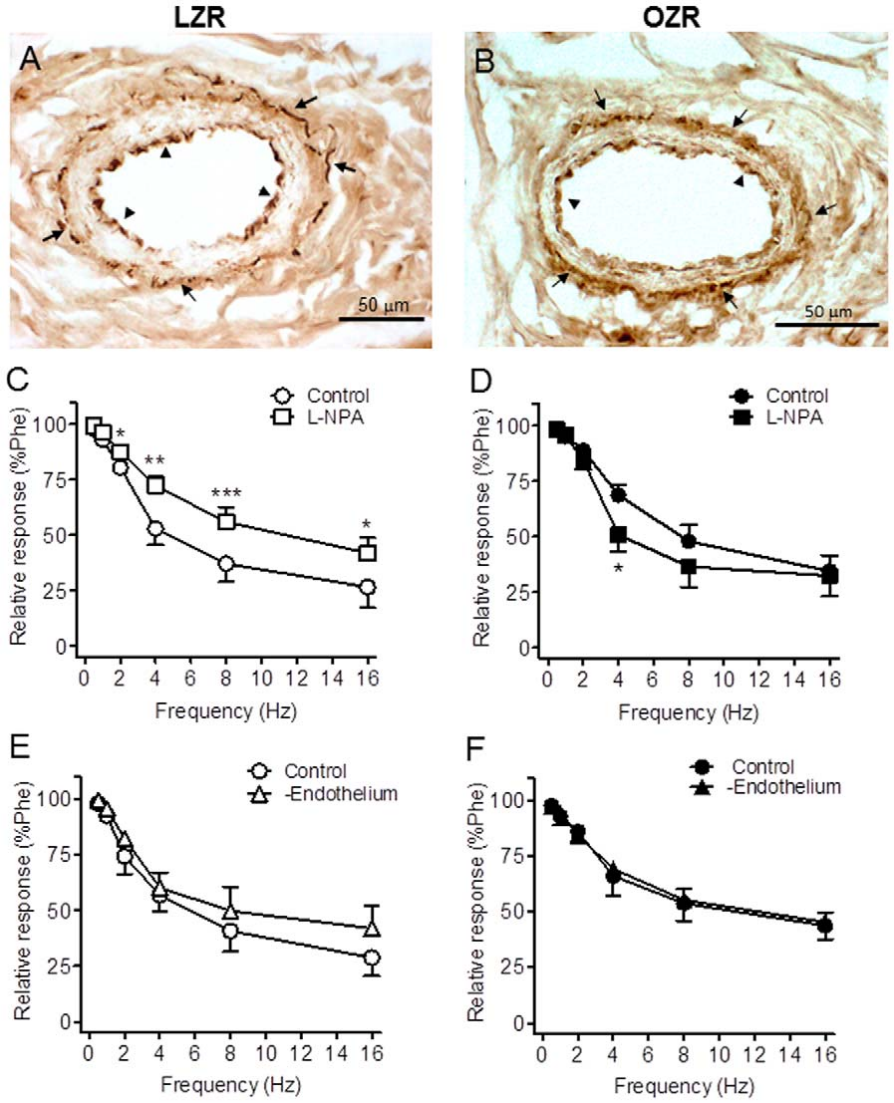
NO derived from nNOS contributes to the nerve-mediated relaxations in penile arteries from LZR but not OZR. **A, B.** Immunohistochemical demonstration of the presence of nNOS in perivascular nerve fibers: in the adventitia (arrows), and in the endothelial cell layer (arrow head) of the penile arteries from LZR and OZR. Note that localization of nNOS immunoreaction was similar in LZR and OZR. **C, D.** Selective inhibition of nNOS with N^W^-propyl-L-arginine hydrochloride (L-NPA, 3×10^−6^ M) reduced the EFS frequency- dependent relaxations in penile arteries from LZR but not OZR.. **E, F.** EFS frequency- dependent relaxations were unaltered in endothelium-denuded penile arteries from LZR and OZR. Results are mean ± SEM of 5–7 arteries. *P<0.05, **P<0.01, ***P<0.001 *vs* control.

Reduced NO bioavailability in the diabetic vascular tissue might be due to reduced NO synthesis due to alterations in either the content or the activity of the enzyme NOS. Immunostaining of cross arterial sections with a polyclonal antibody against nNOS revealed higher nNOS localization not only within the adventitial perivascular nerves but also in the endothelium lining of penile arteries (Figure 4A and B). No differences in either the presence or distribution of this constitutive NOS isoform were observed in penile arteries from OZR compared to LZR rats (Figure 4A and B).

### Contribution of nNOS to the NO-mediated neurogenic relaxations in penile arteries

Selective inhibition of nNOS with L-NPA (3×10^−6^ M) resulted in a significant reduction of the EFS-induced relaxations of penile arteries from LZR (Figure 4C) indicating that nNOS-derived NO contributes to the neurogenic relaxations in healthy penile arteries. In contrast, inhibition of nNOS did not reduce but rather improved the relaxations at low frequencies of EFS in arteries from OZR (Figure 4D), which suggests that nNOS activity might be impaired and the enzyme releases a contractile factor in arteries from obese animals.

Mechanical endothelium removal did not alter the relaxant responses to EFS in arteries from either LZR or OZR (Figure 4E and F), which initially rules out any significant contribution of NO derived from endothelial NOS to the EFS-induced relaxations of penile arteries from OZR and LZR.

### Role of superoxide production in the impaired neurogenic NO-mediated responses of penile arteries from OZR

**Figure 5:**
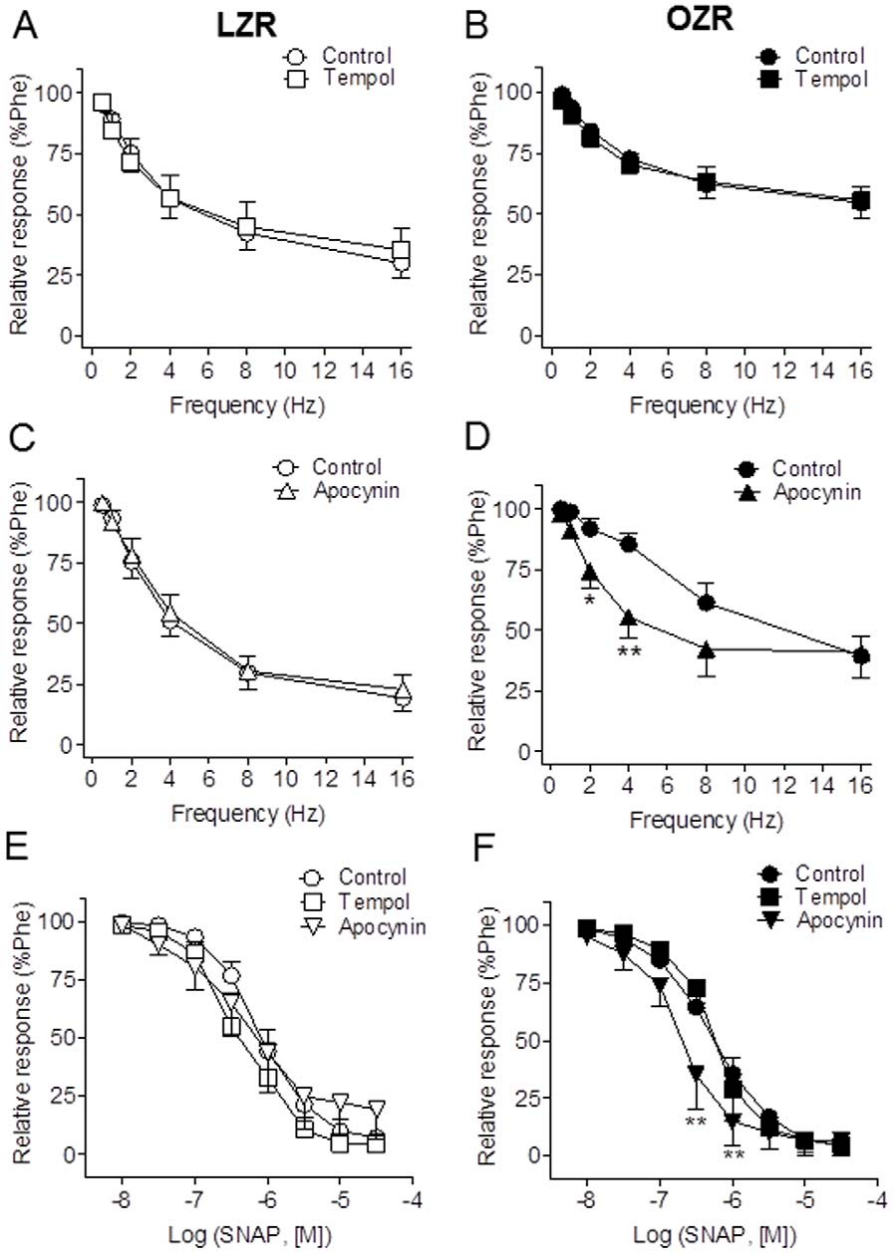
NADPH oxidase inhibition restored the impaired neural relaxant responses in penile arteries form OZR. **A, B.** The superoxide scavenger tempol (3×10^−5^ M) had no effect on either the neural NO-mediated relaxations induced by EFS. **C, D.** Treatment with the inhibitor of NADPH oxidase, apocynin (10^−4^ M) restored the EFS nitrergic relaxation in OZR arteries. **E, F.** Effects of tempol and apocynin on the relaxant responses to the NO donor SNAP (10^−8^–3×10^−5^ M) in penile arteries from LZR and OZR. Results are mean ± SEM of 6–11 arteries. *P<0.05, **P<0.01 *vs* control.

We next investigated whether reduced NO-mediated responses could be the result of increased consumption of NO by ROS. Acute antioxidant treatment by incubating the arteries with the superoxide dismutase mimetic tempol (3×10^−5^ M) did not affect the relaxant responses induced by endogenous neural-derived NO in control arteries (Figure 5A), nor it restored the impaired NO-mediated responses elicited by EFS in arteries from obese animals (Figure 5B). However, treatment with the inhibitor of NADPH oxidase, apocynin (10^−4^ M) to decrease superoxide production significantly improved the EFS nitrergic relaxation in OZR arteries and restored this response to values similar to those in LZR (Figures 5C and D), indicating that an elevated production of NADPH oxidase-derived superoxide inhibits neurogenic relaxations in arteries from obese animals. The relaxations to the NO donor SNAP were unaltered after tempol treatment in penile arteries from LZR or OZR, but improved after apocynin incubation in OZR (Figures 5E and F).

**Figure 6:**
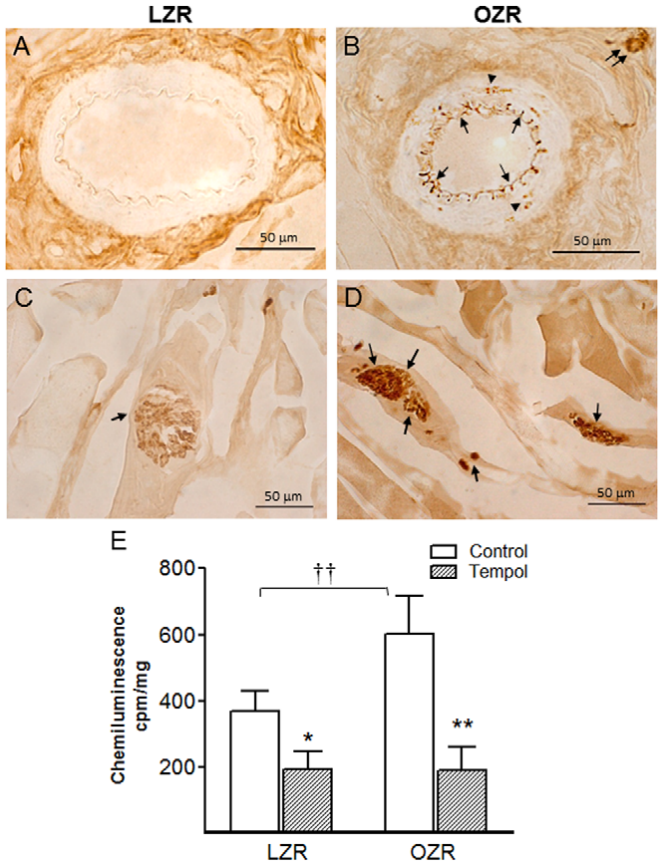
Nitrotyrosine immunostaining for peroxynitrite and superoxide production were elevated in the erectile tissue from OZR. **A–D.** Nitrotyrosine immunoreaction was intense in OZR and was mainly distributed in the endothelial cell layer (arrows) lining penile arteries (B) and cavernosal spaces (D), and eventually in the arterial smooth muscle (arrow heads) and in nerve trunks (double arrows) close to the arteries (B). Nitrotyrosine immunoreaction was slight in penile arteries (A) and corpus cavernosum (C) from LZR. **E.** Basal superoxide production in corpus cavernosum tissue from LZR and OZR detected by lucigenin-enhanced chemiluminescence. Effect of tempol (3×10^−5^ M) on the basal superoxide generated expressed in counts per minute (cpm) per mg of tissue. Data are shown as the means ± SEM of 6–7 corpus cavernosum samples. *P<0.05, **P<0.01 *vs* control. ^††^P<0.01 *vs* LZR.

On the other hand, augmented ROS production in the erectile tissue of obese rats was evidenced in the immunohistochemical study by the enhanced 3-nitrotyrosine staining in sections of both penile arteries and corpus cavernosum. 3-nitrotyrosine immunoreactivity was localized primarily in the inner endothelial layer of penile arteries (Figure 6B) and in the endothelium lining the cavernous spaces (Figure 6D), and could eventually be found restricted to small foci in the media layer of the penile arteries from OZR (Figure 6B). No or slight staining for 3-nitrotyrosine was observed in erectile tissue from control LZR (Figures 6A and C). Measurements of basal superoxide generation by lucigenin chemiluminescence confirmed an enhanced production of superoxide in the cavernosum tissue from OZR compared to LZR (Figure 6E). Preincubation with tempol (3×10^−5^ M) significantly reduced chemiluminescence in erectile tissue from both LZR and OZR corpus cavernosum tissue (Figure 6E), confirming specificity for superoxide (Figure 6E).

### Effect of the NOS cofactor BH4 and the inhibitor of GCH-I on NO-mediated neurogenic relaxations

**Figure 7:**
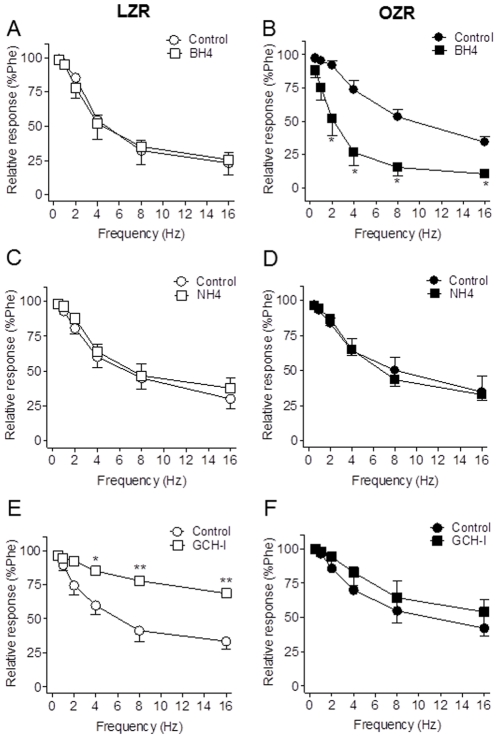
BH4 restored impaired neural NO-mediated vasorelaxation of penile arteries from OZR. Effects of the NOS cofactor BH_4_ (10^−4^ M) (**A, B**) the inactive pterin NH4 (10^−5^ M) (**C, D**). and the inhibitor of the enzyme of BH4 synthesis GTP-cyclohydrolase-I (GCH-I, 3×10^−4^ M) (**E, F**) on the relaxations induced by EFS in penile arteries from LZR and OZR. Results are mean ± SEM of 6–7 arteries. *P<0.05, **P<0.01 *vs* LZR.

Reduced NO bioavailability can be the result of NOS uncoupling to the synthesis of NO due to enhanced oxidative stress and diminished levels of the NOS cofactor BH4. Augmenting vascular BH4 levels by pharmacological supplementation enhances the rate of *de novo* or recycling biosynthesis of NO. Preincubation of penile arteries with BH4 (10^−4^ M) restored neurogenic NO-mediated vasodilatation in penile arteries from OZR to levels similar to those in control arteries (Figure 7B), and had no effect on the LZR EFS-induced relaxations (Figure 7A), suggesting that reduced BH4 might account for the impaired NO neurogenic relaxations in OZR. To exclude any antioxidant effects of BH4, NH4 was used as a negative control, since this pterin shares with BH4 antioxidant effects but has no influence on NOS uncoupling. Addition of NH4 (10^−5^ M) had no effects on EFS-induced relaxations in either LZR or OZR (Figure 7C and D). Incubation with the inhibitor of the enzyme of BH4 synthesis GCH (2,4-diamino-6-hydroxypirimidine, 3×10^−4^ M) significantly reduced the NO-mediated neurogenic relaxations of penile arteries from LZR but not from OZR (Figures 7E and F), thus supporting that impairment of the neurogenic penile relaxations in obese animals might be due to decreased GCH activity leading to reduced levels of the cofactor BH4 and to nNOS uncoupling.

### Changes in NO calcium signalling

**Figure 8:**
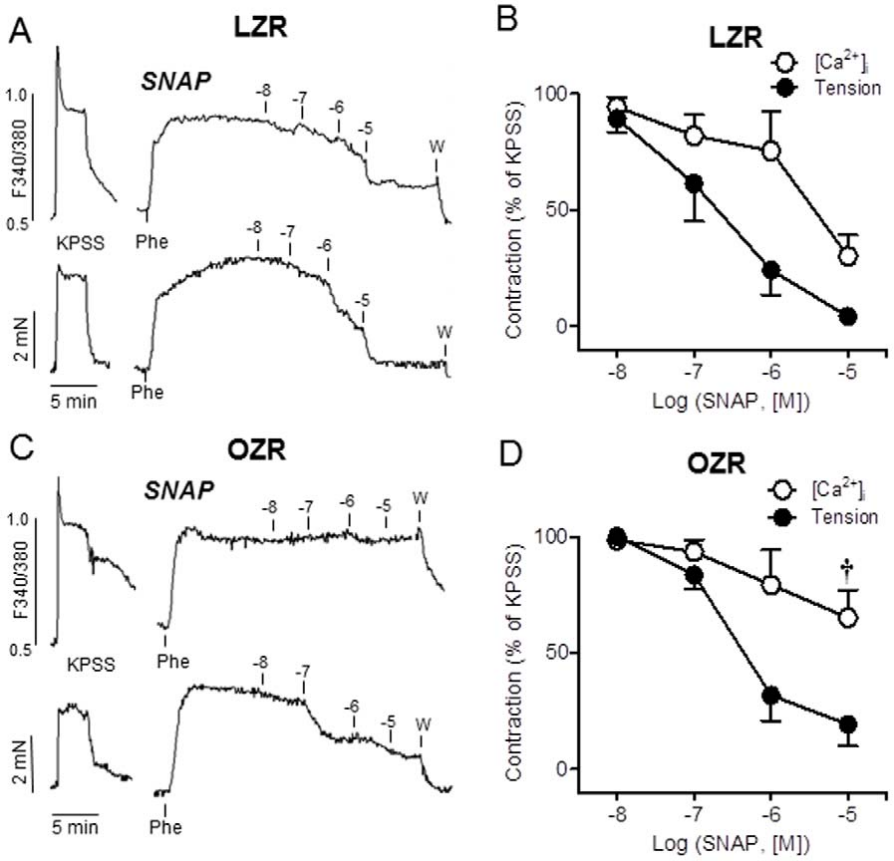
NO calcium signalling is impaired in penile arteries from OZR. **A, C.** Simultaneous recordings of [Ca^2+^]_i_ (top) and tension (bottom) showing the effects of the NO donor SNAP (10^−8^–10^−5^ M) on Phe-precontracted penile arteries from LZR (A) and OZR (C). **B, D.** Summarized data showing the changes in [Ca^2+^]_i_ and force in response to SNAP, and the impaired decrease in the [Ca^2+^]_i_ in response to the highest concentration (10 µM) of SNAP applied in OZR (D). Responses are percentage of the rise in F_340_/F_380_ and contraction elicited by Phe. Results are mean ± SEM of 5–6 arteries. **†**P<0.05 *vs* LZR.

In order to assess whether enhanced oxidative stress could impair the NO signalling pathways of penile arteries from OZR despite the preserved NO-induced relaxations, simultaneous measurements of [Ca^2+^]_i_ and tension were performed in arteries from LZR and OZR. Stimulation of penile arteries from LZR and OZR with high K^+^ solution (KPSS) induced simultaneous increases in both [Ca^2+^]_i_ (Δ(F_340_/F_380_) = 0.23±0.06, n = 5, in LZR, and 0.31±0.05, n = 6, in OZR, *P*>0.05) and tension (1.87±0.50 Nm^−1^, n = 5 in LZR and 1.37±0.19 Nm^−1^, n = 6 in OZR, *P*>0.05) (Figures 8A and C). The α_1_-adrenoceptor agonist Phe (10^−6^ M) also evoked sustained increases in [Ca^2+^]_i_ (Δ(F_340_/F_380_) = 0.19±0.04, n = 5, in LZR, and 0.22±0.03, n = 6, in OZR, *P*>0.05) and tension (2.05±0.47 Nm^−1^, n = 5 in LZR and 1.84±0.28 Nm^−1^, n = 6 in OZR, *P*>0.05) (Figures 8A and C).

The relaxations induced by the NO donor SNAP (10^−8^–10^−5^ M) in arteries pre-contracted with Phe were accompanied by simultaneous decreases in [Ca^2+^]_i_, especially at the highest concentrations of SNAP, in penile arteries from LZR (Figures 8A and B). In contrast, the decreases in [Ca^2+^]_i_ induced by SNAP were impaired in arteries from OZR (Figures 8C and D) while the relaxant effect was not different from that in control arteries (Figures 8C and D). Thus, at the highest concentrations applied (10 µM), SNAP reduced [Ca^2+^]_i_ by 70±9% in LZR, and by 35±12% (*P*<0.05) of the Phe induced contraction in penile arteries from OZR (Figures 8B and D).

## Discussion

Since NO derived from NANC nerves and endothelium is essential for penile erection, dysfunction of the nitrergic system plays a key role in the pathophysiology of vasculogenic ED. The present study demonstrates altered neural NO-mediated relaxant responses due to oxidative stress and nNOS uncoupling in penile arteries from the insulin resistant OZR. This dysfunction likely contributes, along with the endothelial dysfunction also involving altered NO signalling [23], [24], to the decreased erectile responses found in this model of metabolic syndrome/prediabetes [17].

The development of a selective nitrergic dysfunction has consistently been demonstrated in the corpus cavernosum of diabetic impotent men and in the penis of type 1 diabetic animal models [12], [13], [15], [21], while nitrergic neural relaxations were reported to be either deficient [18], [19] or augmented to compensate altered erectile responses [20] in insulin resistance/type 2 diabetic models. The present study demonstrates that the NANC inhibitory neurotransmission is markedly impaired in penile arteries from OZR, which is consistent with the diminished *in vivo* erectile responses to stimulation of pelvic nerves reported in this model of metabolic syndrome [17]. The fact that these neural relaxant responses were largely inhibited by non-selective NOS blockade and that this inhibition was reversed by incubation with the NO synthesis substrate L-arginine, demonstrates that NO is a main neural mediator involved in these nerve-mediated relaxations. On the other hand, the lack of effect of endothelial removal, along with the inhibition elicited by selective nNOS blockade on the EFS-induced relaxations of healthy penile arteries suggest that neurogenic relaxations are mediated by NO originating primarily from neural nNOS. However, selective nNOS inhibition failed to reduce and paradoxically tended to improve the impaired EFS-elicited relaxant responses in OZR, thus indicating that the contribution of nNOS to the neurogenic relaxations is impaired and the enzyme appears to release a constrictor factor in obese animals.

Vascular dysfunction in diabetes includes enhanced vasoconstriction which has been ascribed to either abnormal smooth muscle reactivity or endothelial dysfunction. Sympathetic nerves are responsible for penile detumescence through the release of vasoconstrictor noradrenaline that contracts smooth muscle of arteries and sinuses [5], [6]. The present study shows an enhanced noradrenergic vasoconstriction elicited by nerve stimulation of penile arteries from the insulin resistant OZR, in accordance with the large hypercontractile sensitivity to noradrenergic stimuli associated to decreased erectile responses reported for animal models of insulin resistance/type 2 diabetes [17], [18]. Interestingly, this augmented vasoconstriction seems to be due in part to a dysfunctional nitrergic system, as depicted by the lesser effect of NOS blockade to augment adrenergic contractions in penile arteries from OZR. In LZR, inhibition of NO synthesis greatly enhanced neural noradrenaline-induced contractions thus indicating that NO counteracts noradrenaline responses in healthy arteries and supporting earlier studies showing that nitrergic nerves modulate sympathetic vasoconstriction in human penile erectile tissue [28]. Subsequently, altered NO signalling, as shown in the present study, likely contributes to the impaired erectile responses under conditions of insulin resistance not only by impairing NO-induced vasodilatation but also by augmenting noradrenaline-mediated antierectile effects.

In the present study, reduced neural NO-mediated vasodilator responses were not accompanied by diminished relaxations to the NO donor SNAP in penile arteries from OZR, which initially suggests an intact NO signalling in smooth muscle but impaired NO bioavailability. The latter may be a result of reduced NO synthesis and activity, in turn due to alterations in either the content or the activity of the enzyme NOS. Decreased nNOS expression and activity along with reduced erectile function has been reported in the penis of type 1 diabetic animals [14], [15], [21], [29], where a selective nitrergic degeneration has been demonstrated with loss of nNOS in the nerve fibers and nitrergic dysfunction [15], [21]. In the present study, no apparent changes in the perivascular nNOS containing nerves of penile arteries from OZR were observed, which is consistent with the lack of changes in the nNOS protein content reported for the penis in this model [17] and also in other models of type 2 diabetes [20]. Interestingly, nNOS was also localized in the penile endothelium of both healthy and insulin resistant obese animals, which is in agreement with earlier reports in the corpus cavernosum of diabetic rats [29] and supports functional findings in vascular tissues showing that endothelium- dependent vasodilatations may be at least partially reliant on nNOS-related mechanisms [30].

Enhanced reactive oxygen species (ROS) production is a main factor in the pathogenesis of the vascular complications and coronary artery disease in diabetes, and has been suggested to be involved in the autonomic dysfunction of the rat diabetic heart and penis [31], [32]. Altered NOS function and NO bioavailability has been mostly attributed to vascular superoxide production, NADPH oxidase activation, dysfunctional eNOS and the mitochondrial electron transport chain, being the main sources of ROS generation [33].The findings in the present study suggest that an enhanced NADPH oxidase-derived ROS production is likely interfering with the NO signalling and thus impairing NO-mediated neurogenic relaxant responses in arteries from OZR, as depicted by augmented superoxide levels and the beneficial effect of the NADPH oxidase inhibitor apocynin on the relaxations induced by both exogenous and neural-derived NO in the erectile tissue of obese animals.

Superoxide production by vascular tissues and its interaction with NO might generate the powerful oxidative and highly toxic peroxynitrite radical, that causes oxidative damage to DNA, proteins and lipids, eNOS uncoupling, augmented apoptosis and tissue injury and inflammation [33]. Nitrosative stress induced either directly by metabolic disturbances within the neuron or indirectly by vascular dysfunction has been implicated in the diabetic neuropathy [15], [21], [34]. In the present study, nitrosative stress was demonstrated in the penile erectile tissue from OZR by the enhanced nitrotyrosine immunostaining mainly found in the vascular endothelium of penile arteries, in the endothelium lining the cavernous spaces and eventually in nerve trunks close to arteries and sinuses in the penis of insulin resistant OZR.

Peroxynitrite can oxidize and reduce the availability of the NOS cofactor BH4 and also reduce cellular transport of the eNOS substrate L-arginine, causing eNOS uncoupling. Uncoupled eNOS generates superoxide instead of NO that continues to alter cell signalling processes in vascular endothelial and smooth muscle cells [35], [36], [37]. eNOS uncoupling and endothelial dysfunction have recently been demonstrated in models of hypercholesterolemia- and age-related ED [38], [39]; however, there is limited information of the uncoupled nNOS to cause neural and vascular dysfunction. Our results suggest that dysfunctional nitrergic vasodilator responses in the penile circulation may be due to decreased formation of NO because of uncoupled nNOS. Thus, incubation with the substrate of NO synthesis L-arginine or addition of the NOS cofactor, BH4, restored the nitrergic relaxations induced by stimulation of the nerves in penile arteries from OZR to levels similar to those in healthy arteries. The fact that BH4 addition restored the impaired NO-mediated neural responses in obese animals suggests NOS uncoupling because of relative BH4 deficiency. Moreover, the paradoxical effect of the selective nNOS inhibitor improving the neural relaxant responses along with lack of effect of endothelial removal in arteries from obese animals, further suggests that the neural nNOS isoform is likely to be uncoupled and produces superoxide anions instead of NO, superoxide in turn acting as vasoconstrictor factor.

Since ROS not only directly oxidize the eNOS cofactor BH4 to BH2, but also trigger proteasome-dependent degradation of the rate-limiting enzyme in BH4 synthesis, GCH-I [40], we assessed the effects of GCH-I blockade on the neural relaxations of penile arteries. This treatment significantly inhibited the EFS-induced vasodilation only in LZR but not OZR indicating an altered GCH-I enzyme function as a cause of BH4 deficiency and nNOS uncoupling in OZR. These findings are consistent with the reduced GCH expression recently shown in cerebral arteries from insulin resistant OZR along with the augmented ROS production due to uncoupling of both eNOS and nNOS [41].

On the other hand, the enhancing effect of L-arginine on the impaired vasodilator responses to nerve stimulation of penile arteries from OZR is consistent with earlier studies showing that dietary L-arginine supplementation, as well as acute infusion of L-arginine resulted in an improved NO release and enhanced both endothelium and neural–dependent relaxations in the penis of diabetic and aged rats [42], [43], [44]. Plasma concentration and vascular content of L-arginine are reduced in experimental diabetic animals [45] as well as in diabetic patients [46]. In the present study, the beneficial effect of acute L-arginine administration on the NO-mediated neural relaxant responses initially suggests a deficiency in L-arginine in penile arteries under conditions of metabolic syndrome. Since peroxynitrite can reduce L-arginine transport into the cells by a nitrosative action on the L-arginine transporter CAT-1 [47], oxidative stress and the high levels of peroxynitrite found in the erectile tissue from insulin resistant OZR might explain the acute effect of L-arginine to restore the NO-mediate neural responses.

Finally, the present results demonstrate impaired NO signalling in smooth muscle despite the unchanged relaxations elicited by exogenous NO in penile arteries from OZR. Thus, the NO relaxant responses were accompanied by decreases in intracellular Ca^2+^ in healthy arteries, probably as a result of an activation of Ca^2+^ and/or voltage dependent K^+^ channels and subsequent hyperpolarization [48], [49], whereas in arteries from insulin resistant animals, NO-elicited relaxations were significantly less dependent on changes in intracellular Ca^2+^ suggesting a greater contribution of Ca^2+^-independent mechanisms. The vasodilator action of NO is in part mediated by Ca^2+^ independent mechanisms through an inhibition of RhoA-induced Ca^2+^ sensitization and actin cytoskeleton organization [50]. Therefore, the major involvement of Ca^2+^ desensitization mechanisms in the NO vasodilator action in OZR might be ascribed either to impaired arterial K^+^ channel function by peroxynitrite [51], [52] and/or to enhanced RhoK activity, as demonstrated in the penis of diabetic animals [53] and in the augmented Ca^2+^ sensitization associated to vasoconstriction in penile arteries from insulin resistant OZR [54]. Thus, enhanced RhoK activity due to oxidative stress [55] might be involved in the changes in the NO signalling of erectile tissue under conditions of insulin resistance.

Sustained hyperglycemia, the hallmark of both type 1 and type 2 diabetes, is thought to be the driving force leading to oxidative stress and to the signalling changes which damage penile nerves and endothelium and reduce both nNOS and eNOS activity and NO production. In hyperglycaemic models of insulin resistance/type 2 diabetes, impaired erectile function was accompanied by a slight [18] or nule [20] impairment of the neural NO-mediated relaxant responses, which has led to the suggestion that functionally altered nNOS-mediated signalling does not have much pathophysiologic relevance to the development of ED in these hyperglycaemic models of type 2 diabetes. In contrast, the OZR displays only slight hyperglycemia but an abnormal lipidic profile along with impaired erectile function [17], [23]. The present results first demonstrate impaired NO-mediated neural relaxation of erectile tissue due to oxidative stress and nNOS uncoupling, which along with endothelial dysfunction [23], [24], likely contributes to ED under conditions of metabolic syndrome. Epidemiological studies have identified dyslipidemia as an independent, potentially modifiable factor for diabetic neuropathy [11] and it is also a known risk factor for vasculogenic ED [2]. The present study confirms that a nitrergic dysfunction involving nNOS uncoupling and due to oxidative stress contributes to the pathogenesis of ED under conditions of metabolic syndrome and abnormalities in the lipid metabolism [38].
